# Extended iron phthalocyanine islands self-assembled on a Ge(001):H surface

**DOI:** 10.3762/bjnano.12.19

**Published:** 2021-03-05

**Authors:** Rafal Zuzak, Marek Szymonski, Szymon Godlewski

**Affiliations:** 1 Centre for Nanometer-Scale Science and Advanced Materials, NANOSAM, Faculty of Physics, Astronomy, and Applied Computer Science, Jagiellonian University, Łojasiewicza 11, PL 30-348 Kraków, Poland

**Keywords:** hydrogenated semiconductor, iron phthalocyanine (FePc), scanning tunneling microscopy, self-assembly

## Abstract

Self-assembly of iron(II) phthalocyanine (FePc) molecules on a Ge(001):H surface results in monolayer islands extending over hundreds of nanometers and comprising upright-oriented entities. Scanning tunneling spectroscopy reveals a transport gap of 2.70 eV in agreement with other reports regarding isolated FePc molecules. Detailed analysis of single FePc molecules trapped at surface defects indicates that the molecules stay intact upon adsorption and can be manipulated away from surface defects onto a perfectly hydrogenated surface. This allows for their isolation from the germanium surface.

## Introduction

The development of molecular circuitry requires the preparation of nanostructures isolated from the influence of the underlying substrate. This is of crucial importance for atomic and single-molecule prototypes, but holds also for layered materials. Single-molecule prototypes or molecular nanostructures are often prepared on metals, which usually provide a sufficiently low diffusion barrier for efficient self-assembly and simultaneously allow for in-depth analysis through atomically precise tools from the family of scanning probe microscopes [1–3]. At the same time, however, metallic substrates usually influence the properties of adsorbed molecular species, leading to hybridization, charge transfer, or screening at the interface [4–6]. Also, metallic surfaces may provide relatively weak binding, dominated by van der Waals interactions [7], but the lack of a gap results in broadening and shifting of the molecular resonances. In recent years, it has been proposed to add a buffer layer between the metallic substrate and the molecules of interest [8–9]. This approach allows for the decoupling of the molecules or molecular nanoarchitectures from the metallic substrate and, thus, helps to retain the originally designed properties. Different insulating films have already been applied, ranging from ionic salts such as NaCl [8–9], KCl [10–11], or KBr [12], through oxide [13] or nitride [14] layers to molecular wetting layers [15] and two-dimensional materials, such as graphene [16–17], hBN [11,18], or even organic layers [19]. Recently, it has been proposed that a monolayer of transition metal dichalcogenides, for example, MoS_2_, may play a similar role [4,20–21].

Similarly, it has been reported that the passivation of semiconducting materials, which removes surface dangling bonds and significantly reduces surface reactivity, may also provide a sufficiently insulating layer for an efficient decoupling of molecular structures from the substrate influence. Among such surfaces, hydrogen-passivated Si(001):H [22–23], Si(111):H [24], and Ge(001):H [25–28] surfaces are most commonly mentioned. Iron phthalocyanines (FePc) have been studied on Si(111):H [24] and it was concluded that the molecules are weakly coupled to the substrate. Interestingly, in another study, it has been reported that FePc molecules deposited at room temperature on Si(111):H serve as sources of single Fe atoms and undergo de-metalation [29]. Importantly, hydrogen-passivated Si/Ge surfaces may also act as platforms for nanostructurization by the atomically precise desorption of individual hydrogen atoms and the creation of unsaturated dangling bonds (DBs) or DB systems with predesigned architecture [30–31]. In such a way, different atomic nanostructures could be fabricated in a controllable manner; artificial molecules [32] or surface logic gates [33] could act as examples. Further, such nanostructures may be applied in hybrid systems to couple organic molecules with the underlying surface in a controlled way [34–35], or even provide pivot points for nanoscale rotors [30]. It is also worth mentioning that bringing into practice newly designed nanoscale circuits might be beneficial especially on Ge or Si surfaces, since those semiconductors are at the foundations of traditional electronics. Finally, hydrogen-passivated semiconductors may also provide sufficient isolation for organic molecules to allow for the growth of molecular crystals. It has been already shown that PTCDA molecules form ordered islands on hydrogen-passivated Si or Ge surfaces [36–39]. For instance, it has been shown that on Ge(001):H those molecules form hexagonal islands composed from flat-lying molecules that are sufficiently decoupled from the underlying semiconductor [36]. Vicinal Si(001):H has been applied in order to achieve control over the growth of molecular columns of CuPc molecules [40].

Metal phthalocyanines exhibit useful physical, chemical, and electronic properties. They are considered as promising candidates for practical applications in (opto)electronics and photovoltaics, for instance, in solar cells or transistors [24,41–42]. Moreover, they are ideal candidates to study the influence of the interaction between the central metal atom and the surrounding ligands on the overall properties. Commonly, metal phthalocyanines and their derivatives have been investigated on crystalline metal surfaces [43–57]. However, the need to electronically decouple organic moieties from the underlying substrates directed the attention towards insulators and/or semiconductors. Metal phthalocyanines on semiconducting TiO_2_ surfaces have been frequently studied in the context of a future application in photovoltaics [58–59]. A few of the phthalocyanines with different central metal atoms exhibit magnetic properties [60] and thus attract growing attention.

Having this in mind, we have sublimed FePc molecules on a Ge(001):H surface and studied the formation of molecular nanoislands. Our STM data indicate that FePc molecules stay intact upon adsorption. While single molecules are trapped at surface defects and could be manipulated with the STM tip away from the defects onto the perfectly hydrogenated Ge(001):H surface, the major fraction of the molecules could be found within single-layer islands extending surprisingly far over distances reaching hundreds of nanometers. Within these islands FePc molecules adopt an upright orientation, which is characteristic for substrates weekly interacting with metal phthalocyanines. Our combined scanning tunneling microscopy (STM) and scanning tunneling spectroscopy (STS) measurements indicate that the FePc molecules stay intact upon adsorption on the Ge(001):H surface. The gap measured with STS matches well independently recorded data for weekly coupled FePc molecules. Also, it is in good agreement with optical measurements, indicating a week coupling of FePc located within the islands with the Ge(001):H surface.

## Results and Discussion

### Ge(001):H surface

**Figure 1:**
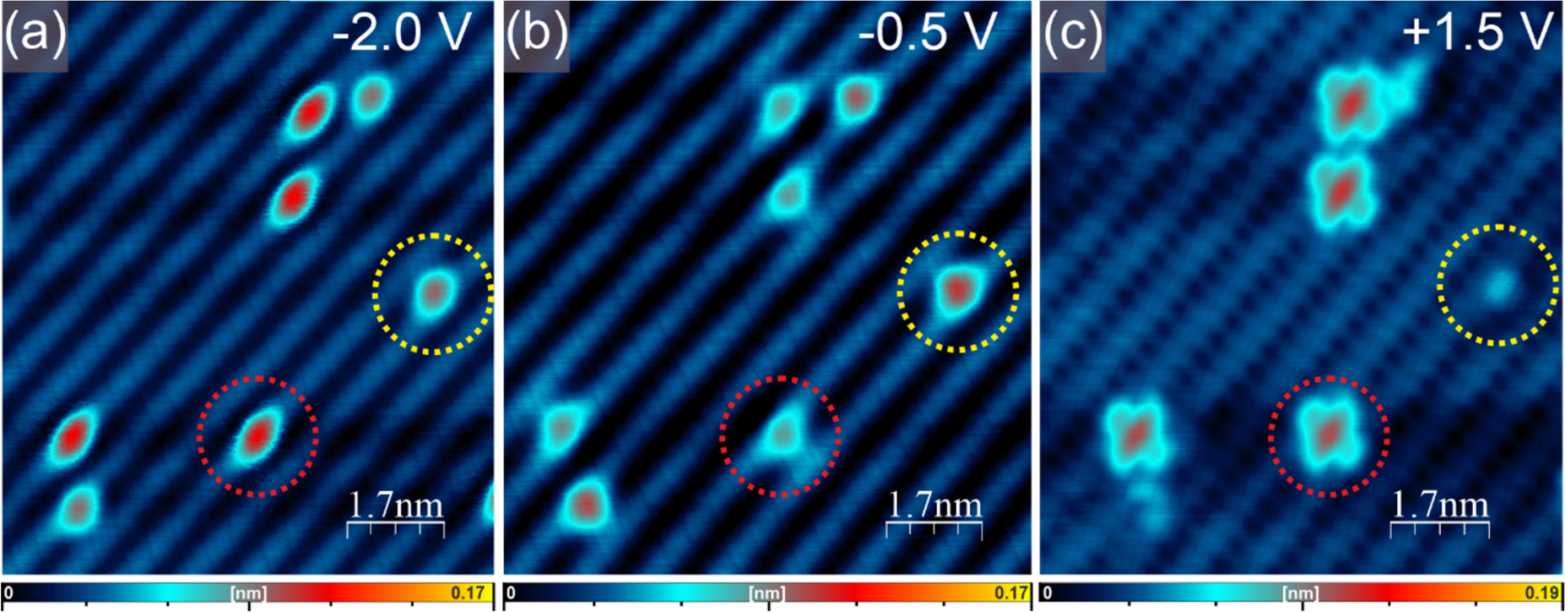
Typical STM appearance of a Ge(001):H surface with atomic-scale defects. (a, b) Filled- and (c) empty-state STM images. The yellow dashed circles indicate a single DB, while the red circles mark a DBD. In panel (c), the characteristic “butterfly” image of the DBD is shown. Imaging conditions: bias voltage −2.0 V (a), −0.5 V (b) and +1.5 V (c); tunneling current: 2 pA.

The Ge(001):H surface exhibits (2 × 1) reconstruction with dimer rows running along the [110]/[1−10] directions. In fact, the hydrogenation is never perfect and some surface defects could be identified within the surface [61–65]. These are mainly single or double hydrogen vacancies. This means that within a Ge dimer either one hydrogen atom is missing, this is called a single dangling bond (DB), or the dimer lacks both hydrogen atoms and the so-called dangling bond dimer (DBD) is formed. A typical STM appearance of the Ge(001):H surface with the above atomic-scale defects is shown in Figure 1.

### Molecular islands of FePc on Ge(001):H

**Figure 2:**
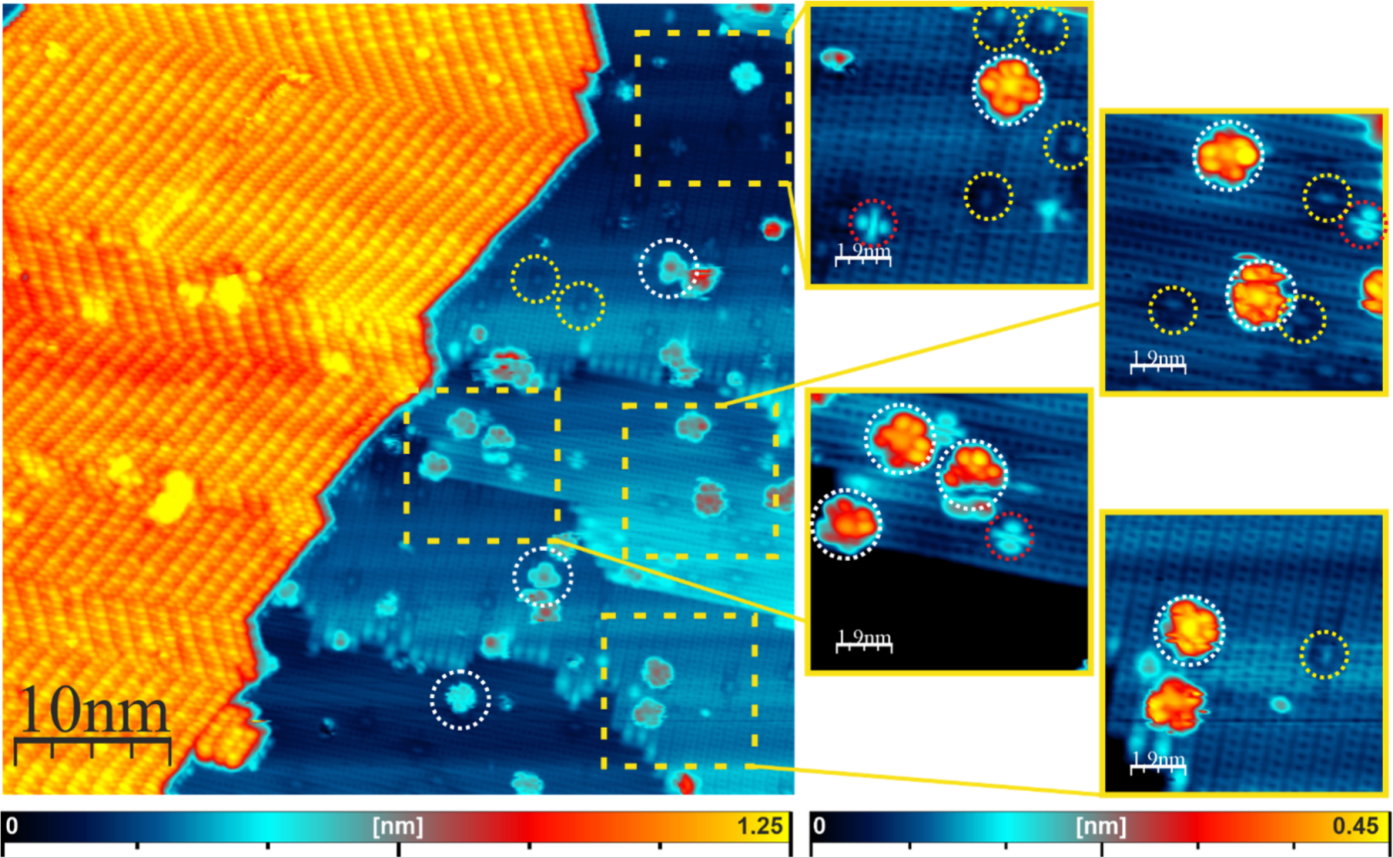
Empty-state STM image of individual FePc molecules and an extended molecular island self-assembled on Ge(001):H. White circles mark individual FePc molecules trapped at surface defects. Yellow circles mark single DBs with a clearly discernible dark halo surrounding them due to single electron charging. Red circles indicate isolated DBDs exhibiting the characteristic “butterfly” appearance. Imaging conditions: bias voltage +2 V, tunneling current 50 pA.

After deposition of FePc molecules onto a Ge(001):H surface at room temperature, we observe single molecules distributed over the surface, as well as extended molecular islands, as shown in Figure 2. Interestingly, the recorded islands partly extend over several hundreds of nanometers, crossing several terrace steps without losing integrity, as shown in Supporting Information File 1, Figure S7. Taking into account the above atomic-scale defects and our previous experiments with other organic molecules [35], we may expect that single FePc molecules on the surface are trapped at surface defects, although the formation of molecular islands hints at a sufficient mobility of the molecules on the Ge(001):H surface. In a previously reported case of starphenes on Ge(001):H, the molecules passivated all DBDs when a sufficient amount of molecules was deposited onto the surface [34]. In contrast to that, we can distinguish unoccupied DBs and DBDs in the vicinity of largely extended molecular islands in case of FePc molecules. This is shown in Figure 2, where single DBs and DBDs are marked, respectively, by yellow and red dotted circles. A number of single FePc molecules, which adopt a flat-lying configuration, could be found on the terraces; for clarity these molecules are marked by white dashed circles.

**Figure 3:**
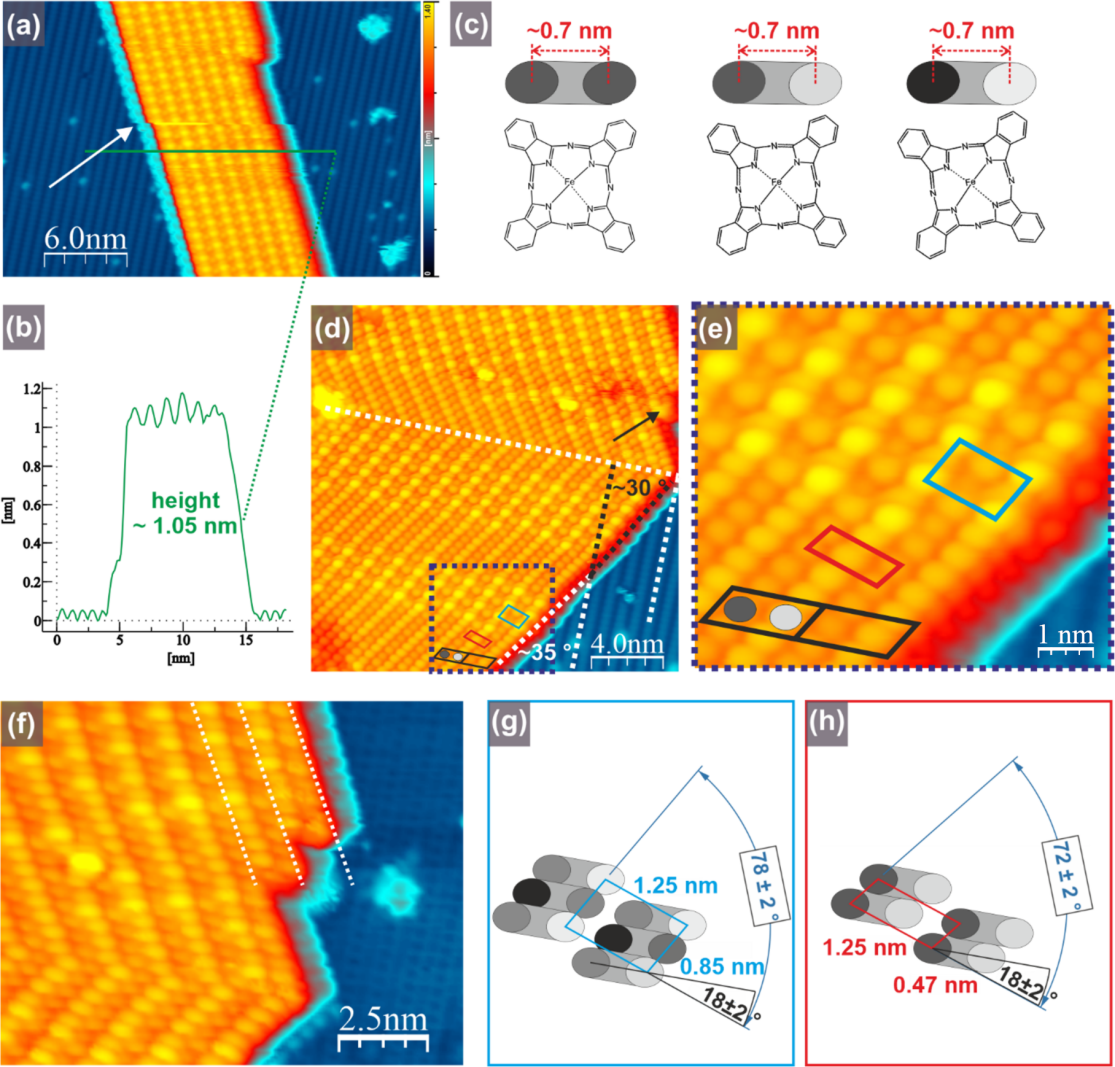
FePc island on a Ge(001):H surface. (a) Filled-state STM image of the island. The white arrow indicates the discontinuity of the FePc island image. (b) Height profile of the FePc island measured along the green line in (a). The apparent height of the island reaches approximately 1.05 nm, which indicates the upright orientation of FePc molecules within the island. (c) Structural scheme of FePc (bottom) with schematic appearance of the STM contrast for upright-oriented molecules within the islands (top view). The differently shaded lobes mimic the contrast variation of the STM appearance due to a slight rotation of the molecules. (d) High-resolution STM image of the CuPc island with clearly visible different domains. One white dashed line indicates the direction of surface-reconstructed rows, while the second white line divides the FePc island into two parts, in which molecular columns are oriented along mirrored directions. Black parallelograms indicate anticipated images of single FePc molecules. Red and blue parallelograms show repeated units of two different domains. The black arrow indicates the place where the island is expanded by one additional FePc column. (e) Magnification of the area marked by a violet dashed rectangle in (d) with unit cells and assignment of the STM appearance of FePc molecules within the island. (f) STM image of the island with clearly noticeable side extension composed of two rows of lobes indicated by the dashed white lines. (g, h) Simplified structural models of different domains indicated in (d) and (e). The variation of lobe contrast mimics differences in the STM contrast. STM imaging conditions: bias voltage −2 V (a, e), tunneling current 100 pA (a, d, e, f).

We begin with the analysis of the molecular islands. First we note that in the case of smaller islands, a slight shift of the FePc island with respect to the underlying Ge(001):H substrate can be seen during scanning; this is shown by a white arrow in Figure 3a. Interestingly, a close inspection of the Ge(001):H surface surrounding the island shows no signs of any discontinuity of the STM appearance. This makes the modification of the STM tip apex unlikely and points to the fact that the observed shift may originate from a real shift of the island on the Ge(001):H surface. This observation indicates a weak interaction between the island and the surface and also a low barrier for island displacement. Further, from the analysis of the apparent height of the molecular island, an upright orientation of the molecules can be inferred. Such a behavior has been frequently reported for substrates on which the interaction between the molecules and the surface is weak. This leads to a dominant role of molecule–molecule interactions and the formation of molecular crystals. In case of phthalocyanines, the upright orientation has been reported, for example, for CuPc on a layer of C_60_ [66] and for CuPc on top of the CuPc wetting layer on TiO_2_ [67]. As indicated in Figure 3b, the STM-measured height of the molecular island reaches approximately 1.05 nm. This is in good agreement with previous reports indicating the STM height of an upright-oriented phthalocyanines to be in the range from 1.10 nm [66] to 1.16 nm [67]. This is much more than a layer of flat-lying molecules, exhibiting an STM height below 0.8 nm [66], and more than the range of 0.3–0.4 nm for single FePc molecules in the present study. In the case of CuPc on TiO_2_ [67], the formation of upright-oriented molecules within assemblies has been achieved by annealing. Here, we obtain islands composed of upright-standing molecules already at room temperature, which indicates a dominant role of intermolecular forces compared to interactions between molecules and hydrogenated surface.

In order to analyze in more detail the properties of the FePc islands, we consider now high-resolution imaging analysis. Already within the STM image in Figure 3a, we can notice lobes that differ in their apparent height. The separation between differently bright nearest neighboring lobes, which reaches approximately 0.7 nm, suggests that they originate from the same molecule. We can introduce a tentative model of the imaging, which assumes that the two unevenly bright lobes correspond to the two outer benzene rings of the same molecule. This is shown schematically in Figure 3c, where the upper panels show schematically the STM top view. The two lobes originating from the very same molecule are colored in different shades of gray. The intensity of the color corresponds schematically to the apparent height of the recorded STM image and, thus, mimic the real height of the specific part of the molecule, that is, the outer benzene ring. This is indicated in the lower part of Figure 3c, where the two outer benzene rings of upright-oriented FePc molecules are always located exactly underneath the corresponding lobes. The actual height of the STM images corresponding to certain benzene rings may arise from a slight rotation of the FePc molecules along the axis perpendicular to the molecular plane, as indicated in Figure 3c. It may also be the effect of a slight variation of the vertical component of the molecule position. However, this does not seem sufficient to provide complete information about the structure of the FePc island. Therefore, in order to obtain more information about the island structure, we consider the image displayed in Figure 3d and the magnification in Figure 3e. Within the image in Figure 3d, the dashed white line indicates the island domain boundary, which is perpendicular to the surface reconstruction rows. The STM image of the molecular island is composed of lobes that are not equally bright. The separation of the neighboring lobes along the dashed white line located at the domain boundary (i.e., across surface reconstruction rows) reaches approximately 0.7 nm, as described above. Close inspection of the island edge at the position marked by the black arrow indicates that the extension of the island is associated with the appearance of two lines of additional lobes. The effect is even better visualized in Figure 3f, where two additional columns of FePc molecules are marked by dashed white lines. This has been repeatedly observed and therefore we can conclude that the island extension is always associated with the appearance of two rows of lobes. Thus, it seems reasonable to assume that the two lobes originate from a single molecule, in accordance with the tentative model of imaging shown in Figure 3c.

For clarity, the anticipated appearance of a single molecule is marked in Figure 3e by a black parallelogram and the two unevenly bright lobes, which are mimicked by two differently colored lobes in Figure 3c. We can notice that the structure of the part of the island located above the white dashed line is almost a mirror image of the part below. We have written “almost” because within both parts of the island one can clearly notice the presence of two subtly different substructures. Their repeating units are highlighted in red and blue in Figure 3e and the corresponding dimensions and angles can be found in Figure 3g and Figure 3h, respectively. The proposed molecule arrangement is indicated by the superimposed anticipated STM images of the molecules containing two lobes for each molecule. While in the “red” structure the neighboring molecules adopt identical orientation and form columns running at approximately 35° with respect to the Ge(001):H surface pattern, the molecules in the “blue” structure form columns at an angle of approximately 30° with respect to the Ge(001):H surface reconstruction rows and exhibit an additional ad-structure. This additional modification comes from the fact that only every second molecule within the row is imaged identically brightly. We mimic this effect by coloring the corresponding STM lobes in different shades of gray in Figure 3g and Figure 3h. In reality, as explained above, this may correspond to a slight rotation along the axis perpendicular to the FePc molecule plane resulting in a non-equal height of the two outer benzene rings, as visualized in Figure 3c. We note here that the uneven brightness of the molecules may also correspond to a slight and periodic variation of the location vertical component of the neighboring FePc molecules. Effectively, the proposed “blue” unit cell is almost twice as large as the “red” one, as a result of the additional contrast modulation described above. In both structures the plane of the FePc molecule is rotated by approximately 18° with respect to the unit cell vector, as visualized in Figure 3g and Figure 3h.

**Figure 4:**
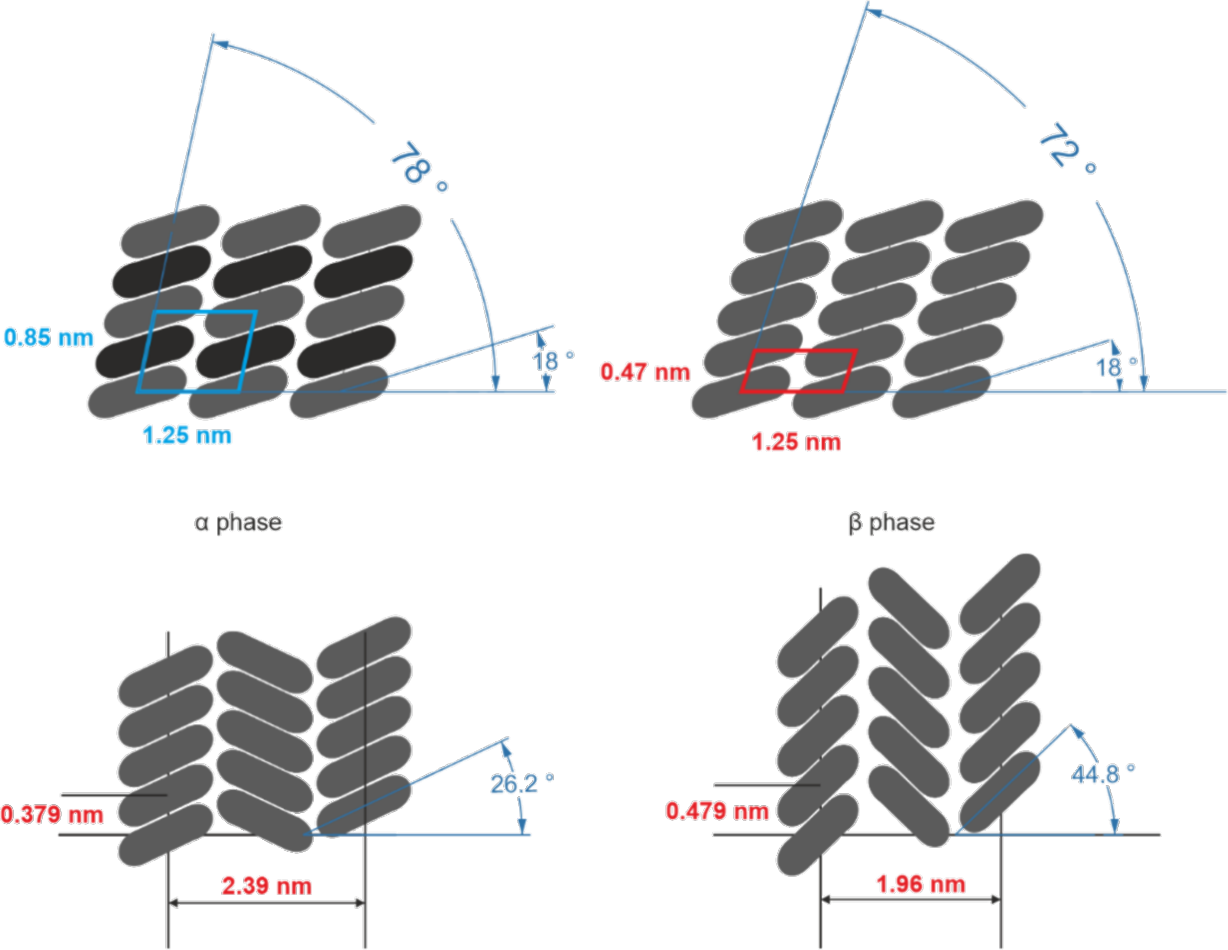
Schematic illustration of the two observed FePc islands on Ge(001):H (top) along with the molecular columns in the α and β phases of FePc (bottom). Images show top views. The differently colored molecules in the “blue” model correspond to molecules differently visualized in STM measurements.

At this point, it is worth mentioning that the observed structure does not correspond to any known FePc crystal phase. This indicates the influence of the substrate–molecule interactions on the crystal formation in the monolayer. The bulk α-FePc phase is characterized by columns within which the separation between the centers of the nearest FePc molecules reaches approximately 3.79 Å [68]. While the neighboring columns contain molecules rotated in opposite directions, the separation between every second column is reported to be 23.9 Å. The β phase is characterized by a slightly larger rotation angle of the molecules within columns, thus resulting in a larger separation between the centers of the nearest neighboring molecules, which reaches 4.79 Å. This is accompanied by a slightly decreased column separation of 19.6 Å for every second column [68]. Nevertheless, our proposed model provides comparable molecule–molecule separations of 4.25 Å and 4.7 Å for the “blue” and “red” structures, respectively. The transversal separation of the columns reaches approximately 1.25 nm, which is slightly more than half of the reported values for the α and β phases. However, our model suggests identical rotation within neighboring columns and, therefore, shall correspond to the half of the unit cells of the α and the β phase. While the majority of phthalocyanines exhibits alternate rotation of the molecules within neighboring columns, there are examples of structures, in which the molecules are rotated uniformly, that is, CuPc on a wetting layer on TiO_2_ [67]. For clarity, the simplified schematic drawings of the α and β phases and our models are shown in Figure 4.

**Figure 5:**
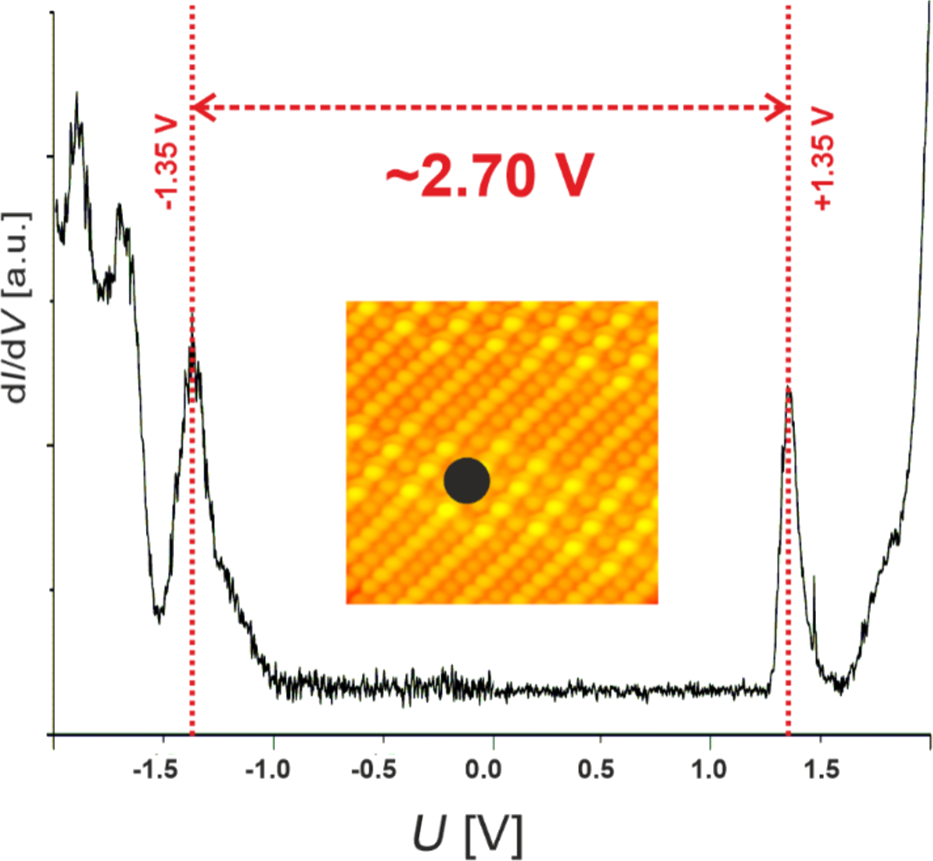
A single-point STS spectrum acquired on a FePc island. The gap measured with STS reaches approximately 2.70 eV, the dot in the inset shows the lateral position of the tip during STS measurements.

In order to acquire information on the electronic properties of the FePc molecules within the islands on the Ge(001):H we have performed STS measurements. Figure 5 shows a single-point spectrum recorded on the FePc island. For clarity, the inset indicates the lateral position of the STM tip during measurements. Within the data we can clearly notice the presence of narrow resonances centered at approximately −1.34 V and +1.36 V, which are separated by a flat part of the spectrum associated with the bandgap of the FePc island. The gap reaches approximately 2.7 eV, which correlates well with the recently reported data for FePc on graphene where the molecules were decoupled from the substrate [17]. Therefore, our results suggest that the FePc islands are well isolated electronically from the influence of the underlying germanium by the passivating hydrogen layer. This is in line with previous reports showing that other organic compounds are well decoupled from the surface by hydrogen, unless they are contacted with the underlying semiconductor through atomic-scale defects, that is, DBs or DBDs [25].

### Manipulation of single FePc molecules

**Figure 6:**
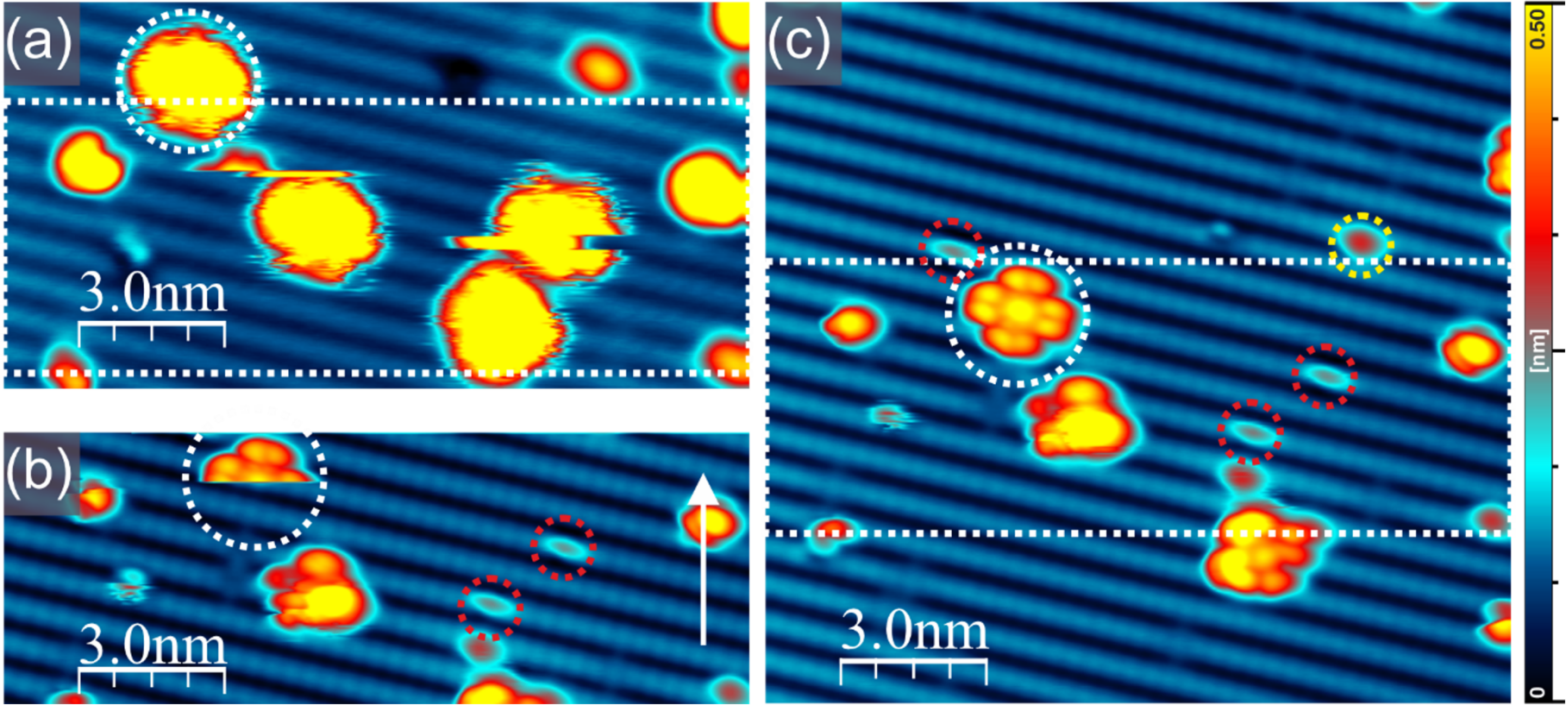
Single FePc molecules trapped at surface defects on a Ge(001):H surface. The white dashed circles indicate the molecule of interest. Molecules visualized in panel (a) are unstable and appear as fuzzy features with clearly discernible lateral displacement events. (a, b) The molecule marked by a white circle is removed from the defect and placed onto a perfectly hydrogenated area. Panel (b) shows the appearance of the molecule during upward scanning. The slow scan direction is marked by a white arrow on the right. (c) High-resolution STM image. The molecule marked by a white circle is located on a perfectly hydrogenated Ge(001):H surface and exhibits the typical appearance of isolated FePc molecules. Different atomic-scale surface defects that can be discerned are marked by dashed circles (i.e., a single DB (yellow) and DBDs (red)). The white dashed rectangles in (a) and (c) indicate the area visualized in (b). Imaging conditions: bias voltage −2.0 V; tunneling current 30 pA.

It is worth noting that the STM appearance of the individual flat-lying FePc molecules is different from that of the molecules in the FePc island. This finding suggests that the molecules are probably trapped at some surface defects and that the interaction is responsible for the modification of the STM-recorded contrast. One can also note that some molecules are clearly unstable during STM imaging. This makes the identification of the FePc molecules uncertain. In order to unambiguously identify single molecules, a single molecule manipulated and moved onto a perfectly hydrogenated Ge(001):H surface. This is shown in Figure 6. Figure 6a shows a few phthalocyanine molecules on the Ge(001):H surface that are trapped at defects and are unstable during STM measurements. This is manifested by their fuzzy appearance. Dynamic behavior of other organic molecules trapped at dangling bonds has been already reported. The DBD located underneath a starphene molecule could, for instance, act as a pivot point of a molecular rotor [30]. The FePc molecule of interest is marked by a dashed white circle in Figure 6. Figure 6b shows the actual moment of unintentional manipulation when the molecule marked by the dashed circle suddenly appeared on a perfectly hydrogenated Ge(001):H surface area. This could be inferred from that fact that the image shows the molecule only in the upper part of the scan, whereas the lower part of the topography presents the perfectly hydrogenated Ge(001):H surface. In a consecutive scan, shown in Figure 6c, the FePc molecule exhibits the typical symmetric appearance. The STM image of the molecule consists of one central lobe located at the anticipated position of the metal atom, which is surrounded by eight lobes. Such an appearance is characteristic for metal phthalocyanines that are isolated from the influence of the substrate, as already shown for FePc on Si(111):H [24] or on graphene [17]. We note here that the image corresponds well to previously reported images acquired at voltages below the values at which resonances on a central atom or the ligands are recorded [24]. The above findings indicate that the FePc molecules stay intact upon deposition on the Ge(001):H surface and that they are decoupled from the germanium substrate by the passivating hydrogen layer. Moreover, the manipulation event allows for the identification of the anchoring sites for the FePc molecules visible within Figure 6a. As visible in Figure 6, the manipulation described above is not the only modification recorded in the scanned area. We can notice two other molecules located in the central part of Figure 6a, which are displaced during scanning and disappear in Figure 6b,c. A comparison of Figure 6a and Figure 6b indicates that all three abovementioned FePc molecules were initially located on defects appearing as narrow bright features in the central part of the Ge(001):H surface reconstruction row. These defects are marked by red dashed circles in Figure 6b,c. A close inspection shows that they exactly resemble DBDs presented in the filled-state STM image in Figure 1a. This allows us to draw the conclusion that the FePc molecules recorded in Figure 6a were immobilized by DBDs. It is worth noting that the preferred localization of polycyclic molecules on DBDs on Ge(001):H has already been reported for starphenes [25,30,34] and tribiphyenylenes [35].

The appearance of the single FePc molecules described above and the fact that we did not record any means of Fe intercalation point to the adsorption of intact molecules. In contrast, on the Si(111):H surface, FePc molecules are reported to lose their central metal atom at room temperature [29].

## Conclusion

We have demonstrated that FePc molecules deposited on a Ge(001):H surface at room temperature self-assemble into extended monolayer islands comprising upright-oriented FePc molecules. The STS data recorded on the islands indicated a transport gap of approximately 2.70 eV, which is in good agreement with previously reported values for isolated molecules. Since the Ge(001):H surface contains atomic-scale defects, a fraction of FePc molecules was found flat-lying and immobilized at these defects. Such molecules could be displaced laterally by means of STM manipulation and placed onto a perfectly hydrogenated Ge(001):H surface, which provided sufficient isolation from the underlying germanium substrate. Based on high-resolution STM images, we have proposed a simplified model of the layer structure, which resembles molecular columns present in phthalocyanine crystals.

## Experimental

The whole experiment was performed in an UHV system equipped with a low-temperature STM manufactured by Omicron Nanotechnology GmbH. The Ge samples used in the experiments were cut from undoped wafers (TBL Kelpin crystals, n-type, 45 Ω·cm). After insertion into the UHV system the samples were sputtered and annealed for 15 min (Ar^+^, 600 eV, 1020 K). The Ge sample was hydrogenated using a custom-built hydrogen cracker following the procedure described in [61]. Deposition of the FePc molecules (Sigma-Aldrich, purity > 99%) was performed from a Knudsen cell manufactured by Kentax. During evaporation the crucible temperature was kept at 330 °C. All STM/STS experiments were performed at liquid helium temperature (ca. 4.5 K) with electrochemically etched Pt–Ir tips used as probes. FePc molecules were evaporated at room temperature.

## Supporting Information

File 1 Additional information on single DB charge state switching on Ge(001):H.
