# Supplementary material for: Extended iron phthalocyanine islands self-assembled on a Ge(001):H surface
Source: Beilstein J Nanotechnol. 2021 Mar 5;12:232–41. doi: 10.3762/bjnano.12.19 (PMC7940817; doi:10.3762/bjnano.12.19)
Supplement: File 1 — Additional information on single DB charge state switching on Ge(001):H. [file Beilstein_J_Nanotechnol-12-232-s001.pdf]

BEILSTEIN JOURNAL OF NANOTECHNOLOGY

Supporting Information

for

Extended iron phthalocyanine islands self-assembled on a Ge(001):H surface

Rafal Zuzak, Marek Szymonski and Szymon Godlewski

Beilstein J. Nanotechnol. **2021,** *12,* 232–241. doi:10.3762/bjnano.12.19

Additional information on single DB charge state switching on Ge(001):H

License and Terms: This is a supporting information file under the terms of the Creative Commons Attribution License (https://creativecommons.org/licenses/by/4.0). Please note that the reuse, redistribution and reproduction in particular requires that the author(s) and source are credited and that individual graphics may be subject to special legal provisions.
The license is subject to the *Beilstein Journal of Nanotechnology* terms and conditions: (https://www.beilstein-journals.org/bjnano/terms)

Charge-state switching of single DBs

At first, on the basis of STM images, we want to demonstrate the initiation of the charge-state switching during filled-state measurements. The process is demonstrated in Figure S1. In Figure S1A (middle part) the filled-state STM image acquired at a low bias voltage of −40 mV is shown. The red arrow indicates the slow scan direction (from bottom to top). At the beginning, the bottom DB is neutral. During approach of the tip it becomes negatively charged. The charging event occurs at the moment when the STM tip is performing the scan line marked by white line. From that moment, both DBs are negatively charged. The charging is visualized by the cross sections shown on both sides of the STM image. The green one recorded for neutral DB exhibits clearly the surface reconstruction rows without any additional distortion. In contrast, the red one prepared for the negatively charged DB shows an additional halo feature, which surrounds the charged DB. When a more negative voltage is applied, the transition between negatively charged and neutral state of the DB is initiated. This is documented in Figure S1B and Figure S1C, showing two STM images acquired at −150 mV and −250 mV, respectively. In both cases a streaky pattern, which corresponds to negative/neutral transitions, is shown. For higher voltages the neutral charge state becomes dominant for single DBs, which is manifested by the disappearance of the streaky features in Figure S1D.

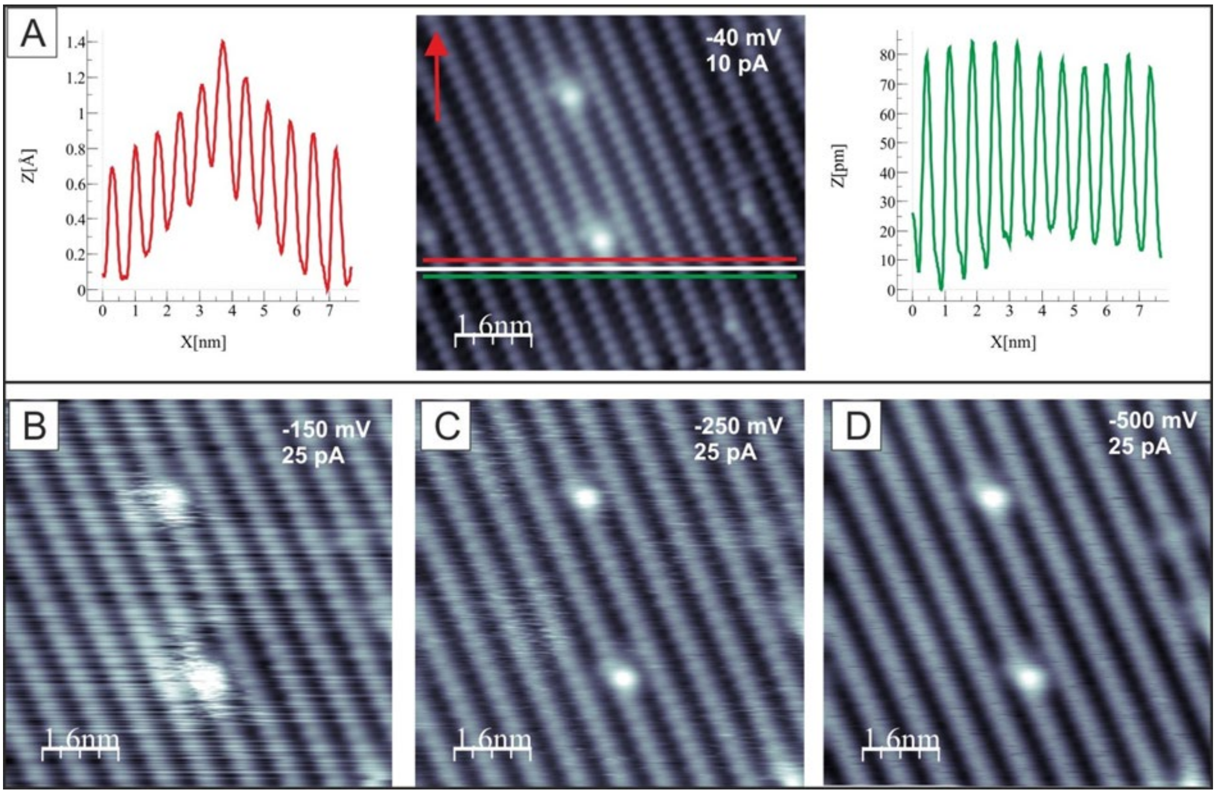

Figure S1: STM images of single DBs. (A) Middle part: filled-state STM image of two single DBs. The white horizontal line indicates the moment of neutral/negative transition of the DB, green and red lines indicate the spatial position of the cross sections presented on left (red) and right (green) sides of the image. The red arrow indicates the slow scan direction. (B, C) Filled-state STM images showing charge transitions of individual DBs. (D) Filled-state STM image acquired with a bias voltage at which the neutral charge state becomes the dominant one.

Following the above experiments we have also performed STS measurements on single DBs. We found that for the tip located directly over the DB, switching between the negatively charged and the neutral state is initiated for voltages in the range from −75 to −250 mV. The values differ slightly between different DBs, but in general for voltages lower (absolute magnitude) than 75 mV all single DBs are negatively charged and for voltages above 250 mV all are neutral. The typical STS $I(V)$ characteristics is shown in Figure S2A. To prove the occurrence of two distinct charge states we have recorded the current-versus-time trace over the DB. This is shown in Figure S2B,

clearly indicating two levels of the tunneling current related to two distinct charge states.

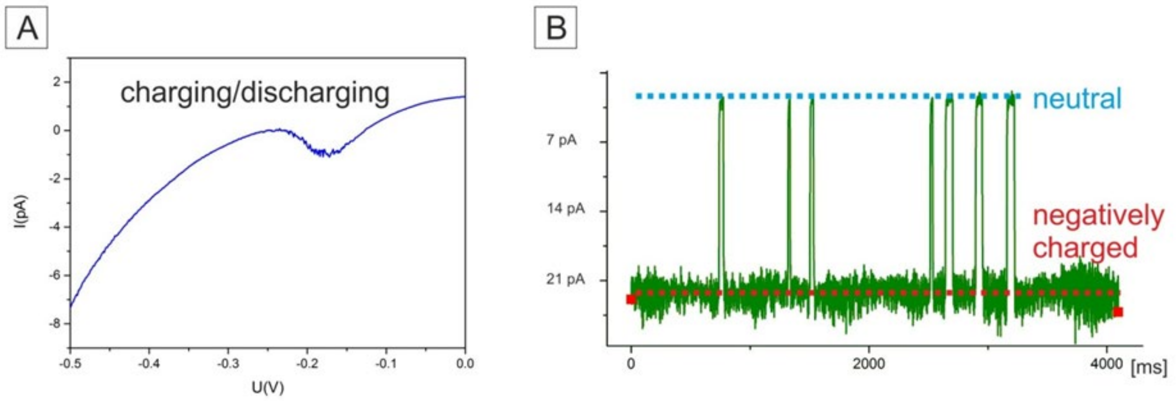

Figure S2: Charging/discharging of the single DB. (A) Typical *I*(*V*) curve. The transition from the negatively charged to the neutral state is observed as the noisy pattern. (B) Typical *I*(*t*) trace recorded on a single DB with a bias voltage of −100 mV. Two distinct current levels corresponding to negative and neutral charge states are clearly seen.

Similar observations could also be made during empty-state imaging. It is well known that the actual charge state of the DB quantum dot depends on the position of the energy levels with respect to the Fermi level and the filling and emptying rates, which describe the injection of electrons (or holes) to the DB from the STM tip and the capturing of the electrons (or holes) by the substrate. In other words, the position of the DB energy levels relative to the Fermi level determines the equilibrium occupation and the filling and emptying rates influence the non-equilibrium state [1], which may fluctuate when these rates are comparable or might determine the non-equilibrium steady state when one of these rates dominates. This means that the current flowing through the tunneling junction may influence the charge state of single DBs. Further, it is important to notice that the voltage applied between the STM tip and the substrate with a low density of states at the Fermi level results in band bending (tip induced band

bending, TIBB). The actual value of the band bending depends on different factors, such as the doping level. For Si(001):H and Ge(001):H surfaces, TIBB may reach up to one third of the applied bias voltage. Summing up, we may say that the charge state of the individual single DBs depends not only on the properties of the sample (i.e., the doping level) but also on the tunneling current flowing through the STM junction and the TIBB.

In STM constant-current imaging, charging of the single DB is recorded as the appearance of a halo surrounding the DB [2-5]. Within the area at which the switching of the charge state occurs with a rate comparable to the scanning velocity the charge transitions could be recorded as the appearance of a streaky pattern. The analysis of the halo around the single DB allows for a precise determination of the actual charge state of the DB. During the empty-state measurements, the appearance of a bright halo indicates positive charging and a dark halo could be assigned to negative charging of the DB. This is because the positively charged DB induces downward band bending, which “helps” to inject electrons to the system. As a result, the STM tip is slightly retracted to maintain the constant current flow. Similarly, a negatively charged DB induces upward band bending around the DB and makes the electron injection “more difficult”. This results in a closer approach of the tip around the DB to maintain the constant current set point and creates the dark halo surrounding the DB. During filled-state imaging, a negatively charged DB induces a bright halo and a positively charged DB creates dark halo surrounding the DB.

STM measurements suggest that on the Ge(001):H surface single DBs are intrinsically neutral. However, when probed with STM with low positive voltage around 0.3 V the single DB becomes positively charged. This is expressed by the appearance of a bright halo surrounding the DB and results from the upward TIBB (Figure S3).

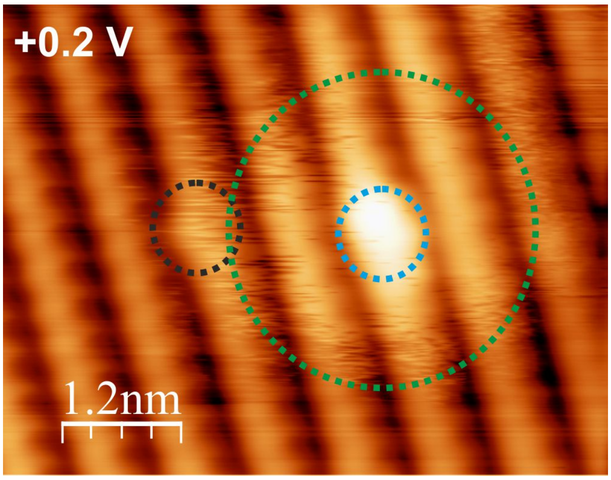

Figure S3: Empty-state STM image showing charge transition of a single DB. The bright feature marked with the blue dashed circle corresponds to the single DB. The black dashed circle indicates the DB dimer. The streaky pattern highlighted in green indicates the lateral position of the STM tip at which the transition to a positively charged state occurs. Tunneling current 10 pA, bias voltage +0.2 V.

The injection of electrons into the DB is very inefficient at low voltages, as the DB state is located outside the energy window available for the tunneling electrons. However, when the bias voltage is increased to the range of 0.4–0.6 V, the electrons start to occupy the DB and switch the charge state to neutral. This leads to the disappearance of the halo surrounding the DB (Figure S4).

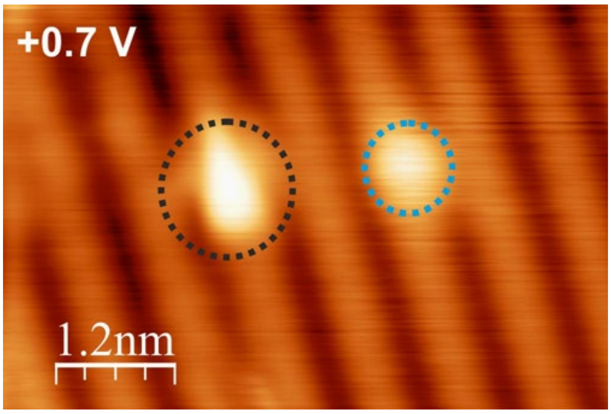

Figure S4: Empty-state STM image showing a neutral single DB marked by the blue dashed circle. The black dashed circle shows a DB dimer. Tunneling current 10 pA, bias voltage +0.7 V.

A further increase of the bias voltage to the range of 0.8–1.0 V results in the injection of a second electron into the DB and makes the DB negatively charged. This is visualized by the appearance of a dark halo surrounding the DB (Figure S5).

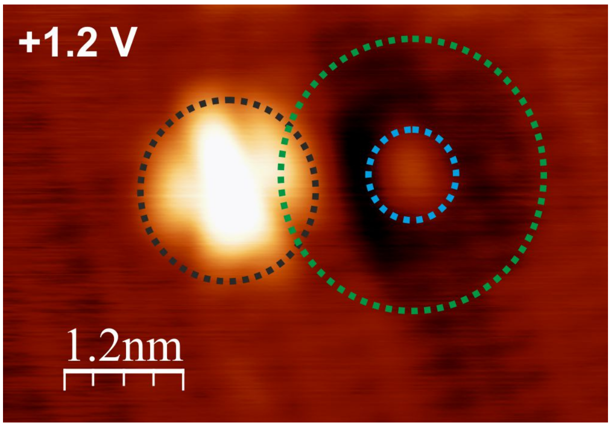

Figure S5: Empty-state STM image showing a negatively charged single DB marked by the blue dashed circle. The green circle indicates the dark halo surrounding the negatively charged single DB. The bright feature marked by the black dashed circle indicates a DB dimer with the characteristic butterfly appearance. Tunneling current 10 pA, bias voltage +1.2 V.

When the bias voltage is further increased, the DB switches back from the negatively charged into a neutral state due to the increased emptying rate, which grows because of the increased TIBB. This is recorded as a streaky pattern observed over the single DB in STM measurements accompanied by the disappearance of the dark halo as (Figure S6). Eventually, as shown in Figure S7, the streaky pattern disappears.

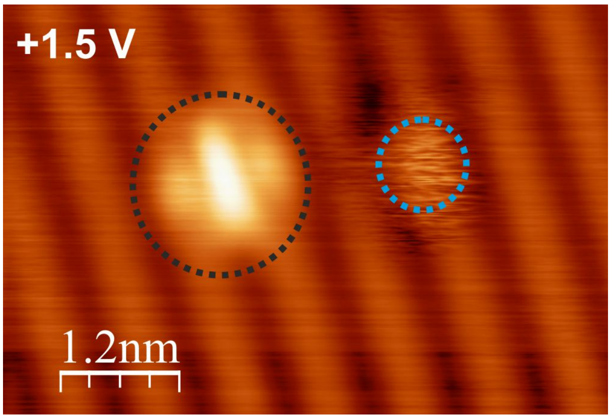

Figure S6: Empty-state STM image showing the charge transition of a single DB. The streaky feature marked with the blue dashed circle corresponds to the single DB. The black dashed circle indicates the DB dimer. Tunneling current 10 pA, bias voltage +1.5 V.

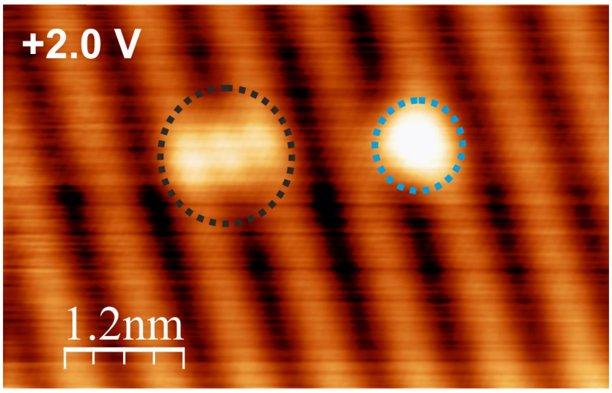

Figure S7: Empty-state STM image showing a neutral single DB, marked by the dashed blue circle. The DB dimer is highlighted by a black circle. Tunneling current 10 pA, bias voltage +2.0 V.

STM imaging of extended FePc islands

In Figure S8 molecular structures that extend over hundreds of nanometers are shown. The molecular nanostructures are elongated along two mutually perpendicular directions, which coincides with the fact that the neighboring terraces of the Ge(001):H surface are characterized by reconstructed rows being oriented perpendicularly.

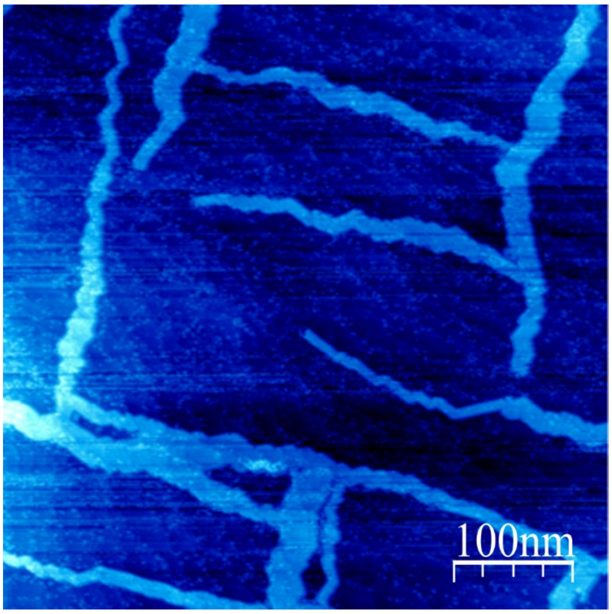

Figure S8: Large-scale STM image of extended FePc islands on Ge(001):H. Imaging conditions: bias voltage −2 V, tunneling current 50 pA.

References

1. Livadaru, L.; Pitters, J.; Taucer, M.; Wolkow, R. A. *Phys. Rev. B* **2011,** *84,* 205416. doi:10.1103/PhysRevB.84.205416
2. Baseer Haider, M.; Pitters, J. L.; DiLabio, G. A.; Livadaru, L.; Mutus, J. Y.; Wolkow, R. A. *Phys. Rev. Lett.* **2009,** *102,* 046805. doi:10.1103/PhysRevLett.102.046805
3. Bellec, A.; Chaput, L.; Dujardin, G.; Riedel, D.; Stauffer, L.; Sonnet, P. *Phys. Rev. B* **2013,** *88*, 241406(R). doi:10.1103/PhysRevB.88.241406
4. Kolmer, M.; Godlewski, S.; Zuzak, R.; Wojtaszek, M.; Rauer, C.; Thuaire, A.; Hartmann, J.-M.; Moriceau, H.; Joachim, C.; Szymonski, M. *Appl. Surf. Sci.* **2014,** *288,* 83–89. doi:10.1016/j.apsusc.2013.09.124
5. Labidi, H.; Taucer, M.; Rashidi, M.; Koleini, M.; Livadaru, L.; Pitters, J.; Cloutier, M.; Salomons, M.; Wolkow, R. A. *New J. Phys.* **2015,** *17,* 073023. doi:10.1088/1367-2630/17/7/073023
